# Supplementary material for: Aldosterone Increases Vascular Permeability in Rat Skin
Source: Cells. 2022 Aug 30;11(17):2707. doi: 10.3390/cells11172707 (PMC9454878; doi:10.3390/cells11172707)
Supplement: Supplementary file 1 [file cells-11-02707-s001.zip › cells-1857611-supplementary.pdf]

cells MDPI

Supplementary Materials

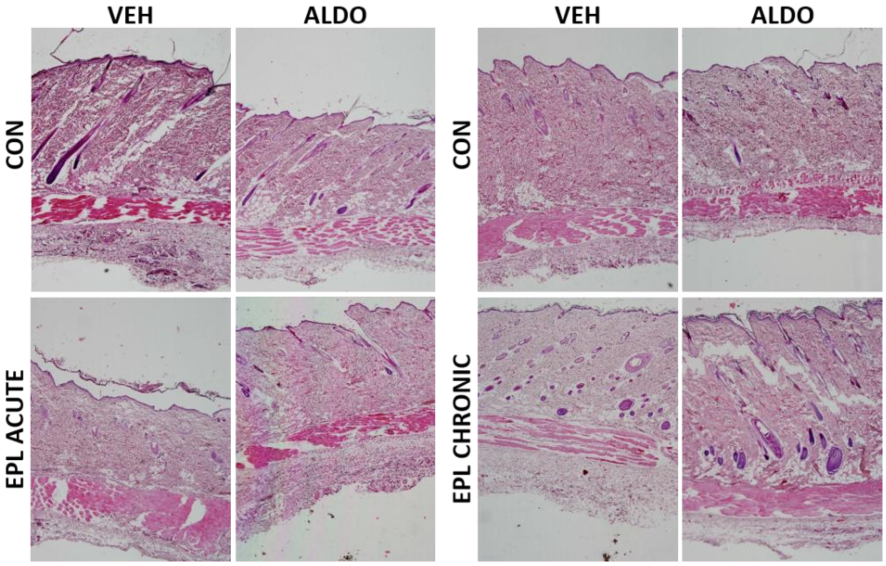

Figure S1. Histological appearance of rat skin tissues with H + E staining. $n = 8$; n for each group is the number of skin samples, not the number of animals; 32 rats were used in this experiment. Magnification ×200. ALDO—aldosterone; CON—5% gum arabic solution; EPL—eplerenone; VEH—ALDO solvent.

Table S1. Percent ratio of total skin thickness for individual skin layers.

Group		Percent of total skin thickness [%]		
		**Epidermis**	**Dermis**	**Hypodermis**
CON	VEH	1.83 ± 0.28	76.15 ± 2.77	22.02 ± 2.81
	ALDO	1.69 ± 0.23	78.39 ± 3.14	19.97 ± 2.94
Acute EPL	VEH	2.49 ± 0.57	78.31 ± 3.33	19.15 ± 3.53
	ALDO	1.79 ± 0.14	79.12 ± 1.82	19.09 ± 1.95
CON	VEH	1.84 ± 0.79	78.22 ± 1.97	19.94 ± 2.12
	ALDO	1.81 ± 0.24	76.57 ± 3.12	19.81 ± 3.07
Chronic EPL	VEH	1.75 ± 0.14	75.89 ± 2.84	22.36 ± 2.86
	ALDO	1.72 ± 0.16	76.56 ± 2.18	21.72 ± 2.24

$n = 8$; n for each group is the number of skin samples, not the number of animals; 32 rats were used in this experiment; data are presented as mean ± SEM; ALDO—aldosterone; CON—5% gum arabic solution; EPL—eplerenone; VEH—ALDO solvent.

Table S2. Blood morphology.

**Group**	**WBC** $n \times 10^3$	**RBC** $n \times 10^6$	**HGB** [g/dl]	**HCT** [%]	**MCV** [µm^3]	**MCH** [pg]	**MCHC** [g/dl]	**PLT** $n \times 10^6$
**CON**	3.38 ± 0.45	8.79 ± 0.21	16.12 ± 0.35	46.08 ± 1.67	52.50 ± 0.96	18.03 ± 0.26	34.58 ± 0.32	655.0 ± 30.96
**Chronic EPL**	3.47 ± 0.17	8.45 ± 0.19	15.80 ± 0.14	46.13 ± 0.53	55.50 ± 1.13	19.69 ± 0.47	34.38 ± 0.18	644.80 ± 11.91

3.6 $n = 6–8$; n for each group is the number of skin samples, not the number of animals; 32 rats were used in this experiment; data are presented as mean ± SEM. CON—5% gum arabic solution; HCT—hematocrit; HGB—hemoglobin; EPL—eplerenone; MCH—mean cell hemoglobin; MCHC—mean corpuscular hemoglobin concentration; MCV—mean corpuscular volume; PLT—platelets; RBC—red blood cells; WBC—white blood cells.
